# Structure and Dynamics of Polymeric Canopies in Nanoscale Ionic Materials: An Electrical Double Layer Perspective

**DOI:** 10.1038/s41598-018-23493-1

**Published:** 2018-03-26

**Authors:** Zhou Yu, Fengchang Yang, Sheng Dai, Rui Qiao

**Affiliations:** 1 Department of Mechanical Engineering, Virginia Tech, Blacksburg, Virginia 24061 USA; 2 Oak Ridge National Laboratory, Oak Ridge, Tennessee 37831 USA; 3 Present Address: JENSEN HUGHES, Inc., Blacksburg, Virginia, 24060 USA

## Abstract

Nanoscale ionic materials (NIMs) are an emerging class of materials consisting of charged nanoparticles and polymeric canopies attaching to them dynamically by electrostatic interactions. Using molecular simulations, we examine the structure and dynamics of the polymeric canopies in model NIMs in which the canopy thickness is much smaller than the nanoparticle diameter. Without added electrolyte ions, the charged terminal groups of polymers adsorb strongly on charged walls, thereby electrostatically “grafting” polymers to the wall. These polymers are highly stretched. They rarely desorb from the wall, but maintain modest in-plane mobility. When electrolyte ion pairs are introduced, the counterions adsorb on the wall, causing some electrostatically “grafted” polymers to desorb. The desorbed polymers, however, are less than the adsorbed counter-ions, which leads to an overscreening of wall charges. The desorbed polymers’ charged terminal groups do not distribute uniformly across the canopy but are depleted in some regions; they adopt conformation similar to those in bulk and exchange with the “grafted” polymers rapidly, hence dilating the canopy and accelerating its dynamics. We understand these results by taking the canopy as an electrical double layer, and highlight the importance of the interplay of electrostatic and entropic effects in determining its structure and dynamics.

## Introduction

Nanoscale ionic materials (NIMs) are a new class of hybrid materials made of nanoparticles and charged polymeric canopies^1–4^. Each nanoparticle carries a net charge, either due to its surface groups or covalently-attached corona featuring ionic functional groups. The canopies, essentially oligomeric counterions, are attached to individual nanoparticles *dynamically* through electrostatic interactions. NIMs are multi-faceted materials: they can be considered as ionic liquids, colloidal dispersions, or composites based on hairy nanoparticles. NIMs have received much attention recently because they exhibit many appealing properties. First, despite that nanoparticles can be tens of nanometers in size and no solvents are used, NIMs are often liquids at room temperature^1^. Second, depending on nanoparticles’ size, shape and composition, NIMs can exhibit a wide spectrum of chemical, optical, and electrical properties. Such an unusual combination of liquid state with the tunable properties of nanoparticles makes NIMs a versatile material platform for diverse applications. Indeed, NIMs have been used as plasmonic material, battery electrolytes, luminescent material, and porous liquids for gas separation^5–9^.

To fully exploit NIMs’ potential for a given target application, their properties must be optimized. Such optimization is not straightforward. Despite their conceptual simplicity, NIMs have vast design parameter space, e.g., the density/charge of nanoparticle’s surface groups/corona, the composition and chain length of the canopy, and the size/shape of the nanoparticles can all be varied. Clearly, optimization guided by knowledge on how design parameters and processing conditions control the structure/dynamics of NIMs and how they in turn determine NIMs’ macroscopic properties, is needed. In particular, a fundamental understanding of the structure and dynamics of NIMs’ canopies is essential. Although research in this area is rather limited, a general picture is emerging.

Many existing studies focused on NIMs made of nanoparticles with moderate/high surface charge density and polymers with relatively long chains^1,5,10^. In these NIMs, the spacing between the charge sites on nanoparticle’s surface (or ionic terminal functional groups of its corona), is smaller than the radius of gyration of *bulk* polymers. As charged polymers are attracted toward the nanoparticle, polymer crowding occurs. An “onion-like” model has been proposed for the canopy in such situations^1,3^: polymers in the canopy form layers around the particle, with those in the inner (outer) layer strongly (weakly) associated with the particle. In neat NIMs, mobility of the polymers strongly associated with the nanoparticle can be more than ten times less mobile than bulk polymers; mobility of the polymers weakly coupled with the nanoparticle, however, is only moderately smaller than that of bulk polymers. The exchange between these populations of polymers is slow^3^. When electrolyte ions are presented in NIMs (either introduced intentionally or exist as contaminants), they screen nanoparticle’s charge. This decreases (increases) the number of the strongly (weakly) associated polymers and facilitates the exchange between polymers in these two populations. When enough electrolyte ions are introduced, the population of the strongly associated polymers diminishes and all polymers exhibit mobility similar to that in bulk^3^. These seminal works greatly improved our understanding of the polymeric canopies, but some important practical questions and conceptual issues remain to be addressed.

From a practical perspective, while the existence of multiple layers (populations) of polymers across canopies is established, many key questions on the quantitative aspects of canopy remain unanswered. Specifically, how are polymers distributed across the canopy – do they form two or more layers? What is the relative population of the polymers in different layers and how does it respond to the addition of electrolyte ions? What conformation do polymers in different layers adopt? Since polymers strongly associated with nanoparticles experiences crowding, their conformation should differ from that of the bulk polymers. However, the conformation of these polymers is not yet known. Hence, the radius of gyration of bulk polymers is often used to infer the packing and thus population of polymers near nanoparticles. The accuracy of this approach is yet to be clarified.

On a more conceptual front, the interactions between the charged terminal group of polymers and nanoparticles were treated or discussed in the framework of “ionic bonds” in most prior studies, which emphasizes the electrostatic “association” between the *individual* charged sites of oligomeric counterions and the *individual* charged sites on the nanoparticles. While this framework has its merit and advantage, the long-ranged nature and global effect of electrostatic interactions is not fully taken into account. Since the charged polymeric canopy and the nanoparticle’s surface charges form an electrical double layer (EDL)^1^, and long-range electrostatic forces play a crucial role in determining the structure of EDLs, the long-range nature of electrostatic interactions warrants attention in studies of canopies. In particular, how long-range electrostatic interactions and polymeric interactions jointly control the structure and dynamics of canopies should be examined.

The above open questions and issues can be addressed using molecular dynamics (MD) simulations. A few such simulations have been reported^11–14^. A common feature of these simulations is that the radius of the nanoparticles is small (~1 nm), the chain length of the polymers is short and comparable to the nanoparticle’s radius (e.g., the linear polymers used in ref.^12^ have ~14 monomers), and the nanoparticles carry moderate surface charge density (e.g., −2 *e*/nm^−2^). Study of these systems revealed that the charged layer of the nanoparticles approaches each other closely and the charged site of polymers can bridge neighboring nanoparticles^12^. The charged site of most polymers are dynamically attached to the charged sites of nanoparticles: a polymer can detach from its original attachment point on a nanoparticle over tens of nanoseconds and become attached to another charged surface site on the same nanoparticle; a polymer can “hop” to another nanoparticle over hundreds of nanoseconds because its charged site can bridge the two nanoparticles. These studies offered useful insights into the structure and dynamics of NIMs. Nevertheless, some important parts of NIM’s parameter space were not explored and the structure and dynamics of the polymeric canopy can exhibit new physics not studied in these studies.

In many NIMs, the nanoparticles have a diameter of a few tens of nanometers and the polymeric canopies are several nanometers thick^2,3,5,15–18^. The surface charge density of nanoparticles can reach ~−4 *e*/nm^2^ in many systems^5^. The thickness of the polymeric canopies and the radius of nanoparticles in these NIMs are thus much larger than those in prior simulations. These have several implications. First, the surface charge layers of adjacent nanoparticles cannot approach each other closely as in the prior simulations and bridging of adjacent nanoparticles by the charged site of the polymers is unlikely. Second, the packing of the polymers dynamically “grafted” to the nanoparticle faces more entropic and steric costs because each “grafted” chain occupies more volume (due to the longer chain length) and space for packing a polymer grafted to the surface of a sphere decreases as its radius increases. Third, the collective, long-range electrostatic interactions become more important not only because the surface charge density of nanoparticles is higher, but also because the collectively screening of a nanoparticle’s surface charge by ions near it becomes less effective as the nanoparticle’s radius increases (This can be seen qualitatively from decay of electrical potential near a charged, isolated sphere in an 1:1 electrolyte ϕ(r) = ϕ_0_a(κr)^−1^e^−(r − a)κ^, where ϕ_0_ is the potential on the sphere’s surface, *a* is the sphere’s radius, and κ^−1^ is the Debye length of the electrolyte, and *r* is the distance from the sphere’s center). Because of these factors, the competition between the electrostatic attraction of charged site of the polymers to the nanoparticles’ surface and the steric and entropic penalties of packing polymers near the nanoparticles can differ from that studied in prior simulations. Consequently, the structure and dynamics of the polymeric canopies in these NIMs can differ from those revealed in prior simulations. Furthermore, in practical NIMs, there are inevitably some contaminant ions, but how such impurity affects the structure and dynamics of the polymeric canopies has not been studied in prior simulations.

In this work, we use MD simulations to study the structure and dynamics of the canopies of model NIMs with relatively long polymer chains, relatively high surface charge density, and in the limit of large nanoparticle diameter. We also investigate how ion contamination affects the properties of canopy. We show that, while the canopy exhibits the onion-like structure proposed earlier, the conformation of polymers strongly associated with the charged walls and the distribution of weakly associated polymers exhibit new features. The EDLs formed by canopies exhibit commonalities with those in room-temperature ionic liquids (RTILs) but also new features unique to NIMs. We show that these observations originate from the interplay of electrostatic and entropic effects in the canopies.

## Simulation System and Methods

### Model systems

**Figure 1:**
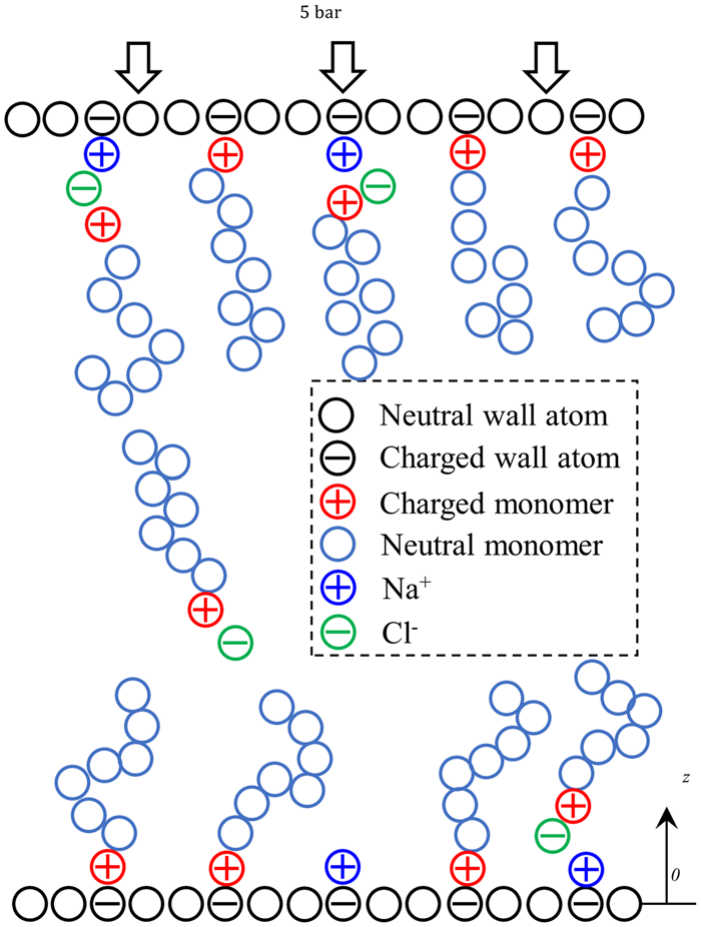
A schematic of the molecular system for studying canopies in corona-free nanoscale ionic materials.

Ideally, a large number of nanoparticles and their polymeric canopies should be included in molecular systems for studying NIMs. While this approach is viable for NIMs with very small nanoparticles (e.g., 1 nm in radius), it is impractical when the nanoparticle diameter is large (e.g., tens of nanometers). Since our focus is the polymeric canopy in NIMs with large nanoparticles, we adopt the model shown in Fig. 1. The system consists of two planar walls with negative surface charge densities, polymers with one of their end monomers carrying a positive charge +*e*, and optionally, pairs of electrolyte ions (to model the electrolyte ions presented in NIMs). The system used here effectively models the canopies between two very large nanoparticles, with the two planar walls representing the surface of each nanoparticle. Hence our model is appropriate only when the curvature of the nanoparticles in NIMs can be neglected, i.e., if the canopy thickness is much smaller than the radius of the nanoparticles. Since the canopy thickness is often a few to tens of nanometers in practical NIMs, our results are relevant to NIMs with nanoparticles measuring tens to hundreds of nanometers in diameter.

We study the canopies in two systems. In the first system, the number of polymers in the canopy is chosen such that their charge exactly balances the charge on the solid wall. The second system has the same number of polymers as in the first system, but electrolyte ion pairs (counterions and co-ions) are added into the canopy. The number of counterions is chosen such that their charge balances 75% of the wall charge. The second system is experimentally relevant since electrolyte ions are inevitably presented in NIMs and sometimes even intentionally introduced to tune NIMs’ properties^3^. Hereinafter, the first and second system will be referred to as neat canopy and doped canopy, respectively. We also simulate a bulk polymer melt that contains the same polymers as in the above two systems except that the charge on each polymer’s end monomer is removed. Table 1 summarizes the setup of the three systems examined in this work.

**Table 1:** Setup of the simulation systems studied in this work.

System	Surface charge density of wall (*e*/nm^2^)	Number of polymers	Degree of polymerization	Number of Na^+^/Cl^−^ pairs
1 (neat canopy)	−3.5	448	25	0
2 (doped canopy)	−3.5	448	25	336
3 (bulk polymer melts)	—	100	25	—

### Molecular models

The solid wall is modeled as a square lattice of Lennard-Jones (LJ) atoms, and measures 8 × 8 nm^2^ in the lateral direction. The lower solid wall is fixed during the simulation. The top wall is modeled as a rigid body. Randomly selected wall atoms are each given a charge of -*e* to produce a net surface charge density of −3.5 *e*/nm^2^ (1*e* = 1.6 × 10^−19^ C), which is within the range of surface charge densities reported experimentally^3,5,19^. A modest external pressure (5 bar) is applied on the top wall to mimic the dispersive interactions between neighboring nanoparticles (see Fig. 1), and we verified that the results changes little if a different pressure (e.g., 10 bar) is applied. Each polymer is modeled as a linear chain of 25 beads bonded by harmonic springs. One of its end beads is given a charge of +*e*, and is hereinafter termed “charged end monomer”. The chain length of the polymers is within the range found in prior experiments^3,5,16^. The force field parameters of the polymer beads are taken from ref.^12^ so that each bead approximately represents one ethoxy repeat unit (-CH_2_OCH_2_-). Briefly, for the bead-bead LJ potential, the depth of its energy minimum is ε_lj_/*k*_B_ = 377 K (*k*_B_ is the Boltzmann constant) and the bead-bead separation at which potential energy is zero is *σ*_*lj*_ = 0.4 nm^12^. Each bead has a mass of 44 g/mol. To account for polymers’ polarizability, a dielectric constant of *ε*_*r*_ = 10 is used in the calculation of electrostatic interactions. This value is on the high end of the dielectric constant for polymers and is adopted so that the desorption of polymer’s charged end monomer from the charged wall can be probed at the time scale accessible in simulations. Without losing generality, Na^+^ and Cl^−^ ions are used to mimic the electrolyte ions presented in NIMs. No solvents are explicitly included because, even in NIMs spiked intentionally by electrolytes, solvents are usually removed by intensive drying^3^. Force fields for the wall atoms and the electrolyte ions are taken from the OPLS-AA force fields^20^, and are summarized in the Supplementary Information.

### MD methods

Simulations are performed using the Gromacs code^21^. The neat and doped canopies (systems 1 and 2 in Table 1) are simulated in the NVT ensemble. The box length in the direction normal to the wall is 50 nm. The position of the upper wall is *not* fixed. The separation between the two walls depends on the canopy’s conformation and is ~10–15 nm for the canopies studied here (see below). The large vacuum space above the upper wall allows the periodicity in the direction normal to the wall to be removed. The polymer melts (system 3) are simulated in the NPT ensemble (P = 1 atm). In all simulations, the temperature is maintained at 400 K using the velocity rescaling thermostat^22^. The elevated temperature chosen here helps to achieve good statistics of polymer displacement. The Parrinello-Rahman pressure coupling scheme is used in the NPT simulations. A time step size of 2 fs is used. The non-electrostatic interactions are computed using direct summation with a 1.0 nm cutoff length. Electrostatic interactions are computed using the PME method (real space cutoff: 1.0 nm; FFT spacing: 0.12 nm). To remove the periodicity in the direction normal to the wall, the slab correction to the PME method is applied^23^. Periodical boundary conditions are applied in the lateral (*xy*−) directions.

To set up systems 1 and 2, 448 polymers and appropriate number of Na^+^/Cl^−^ ion pairs are packed between the two charged walls randomly using Packmol package^24^. Note that the initial chain conformation of the packed polymers is taken from equilibrium runs of polymer melts and thus is not stretched. Systems 1 and 2 are equilibrated for 100 ns and followed by a production run of 400 ns. The evolution of the conformation of representative polymer chains in system 1 is shown in Figure S1 in the Supplementary Information. To set up system 3, 100 polymers with random chain conformation are packed into a cubic box. The system is equilibrated for 30 ns and followed by a production run of 100 ns. Each of the above systems is simulated twice with different initial polymer configurations and the difference in the results is small.

### Data availability

The datasets generated during and/or analysed during the current study are available from the corresponding author on reasonable request.

## Results and Discussion

### Canopy structure

#### Neat canopies

**Figure 2:**
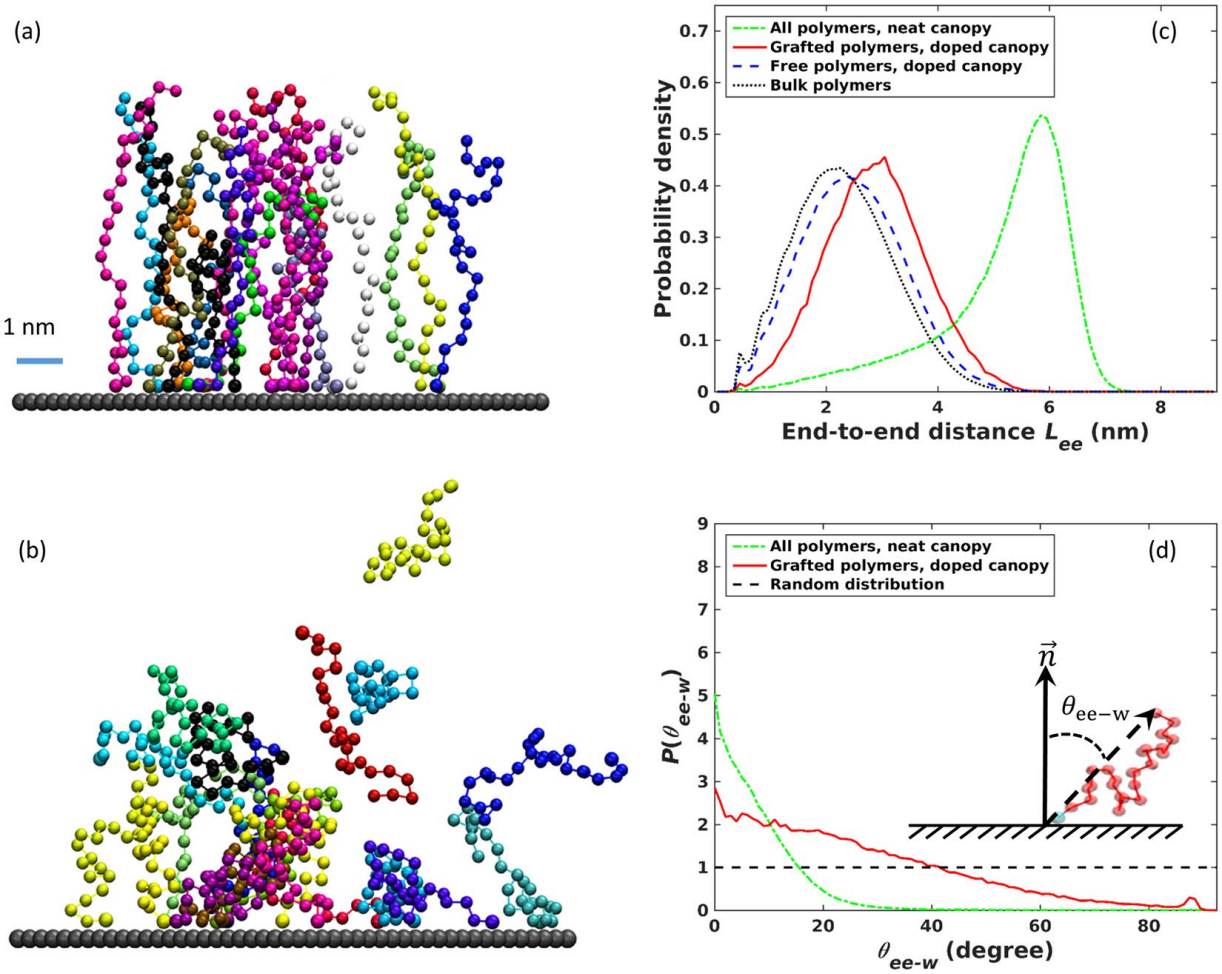
Structure of neat and doped canopies near the lower wall. (**a**,**b**) Snapshots of some representative polymers in the neat (**a**) and doped canopies (**b**). (**c**,**d**) Probability density distribution of the end-to-end distance (**c**) and the angle between end-to-end vector and the normal vector of the charged solid surface (**d**) in neat and doped canopies near the lower charged wall. In (**d**), the probability density is normalized such that a random distribution gives unity at all angles.

We first focus on the conformation of *individual* polymers in the canopies. Here, the positively charged end monomers of nearly all polymers are adsorbed on the negatively charged walls after 10 ns, thereby “grafting” these polymers to the wall. Because of the symmetry of the system with respect to the middle plane between the two walls, hereinafter we focus on the polymers near the lower wall. Figure 2a shows that, at such a high “grafting” density, polymers are highly stretched. Table 2 further shows that, compared to bulk polymers, the mean radius of gyration (*R*_g_) and end-to-end distance (*L*_ee_) of the polymers in the canopy increases by ~75% and ~110%, respectively. The distribution of the polymers’ *L*_ee_ shifts toward their full contour length (see Fig. 2b) and exhibits a sharp peak at *L*_ee_ = 5.85 nm. The population of polymers with *L*_ee_ smaller than 2.48 nm (the mean *L*_ee_ of bulk polymers, see Table 2) nearly vanishes. Hence, the neutral end monomer of these polymers can hardly access the space within ~2–3 nm from the wall. Indeed, Fig. 2d shows that angle formed by the polymers’ end-to-end vector and the wall’s normal vector (*θ*_ee-w_) is restricted to *θ*_ee-w_ < 30°.

**Table 2:** Conformation characteristics of individual polymers in canopies and bulk polymer melts.

	Polymers in the neat canopy	Wall-associated polymers in the doped canopy	Detached polymers in the doped canopy	Bulk polymer melts
Radius of gyration (nm)	1.75 ± 0.00	1.11 ± 0.02	1.01 ± 0.01	1.00 ± 0.02
End-to-end distance (nm)	5.31 ± 0.02	2.99 ± 0.07	2.50 ± 0.06	2.47 ± 0.09

The stretched conformation of the polymers shown in Fig. 2 are in contrast to the more random-coil like chain conformation revealed in the prior simulations^12^. We note that, in both the prior and the present simulations, all the charged monomers are grafted to the charged solid surfaces. However, in the present case, where the surface charge density is ~90% higher than that in ref.^12^, the grafting density of the polymer is much larger. The higher grafting density is compounded by the fact that our polymer chains are ~80% longer than those in ref.^12^. Furthermore, even at the same grafting density (i.e., the number of polymers grafted per unit area of solid surfaces), the packing of polymers grafted on a planar wall (or the surface of a large sphere) is more susceptible to crowding than those grafted on a small sphere because of the smaller curvature of the surface. Because of these factors, polymers are much more stretched in our system than in earlier simulations. This difference highlights the fact that the polymeric canopies in NIMs can exhibit rich structure depending on the choice of material parameters including the particle size and surface charge density.

**Figure 3:**
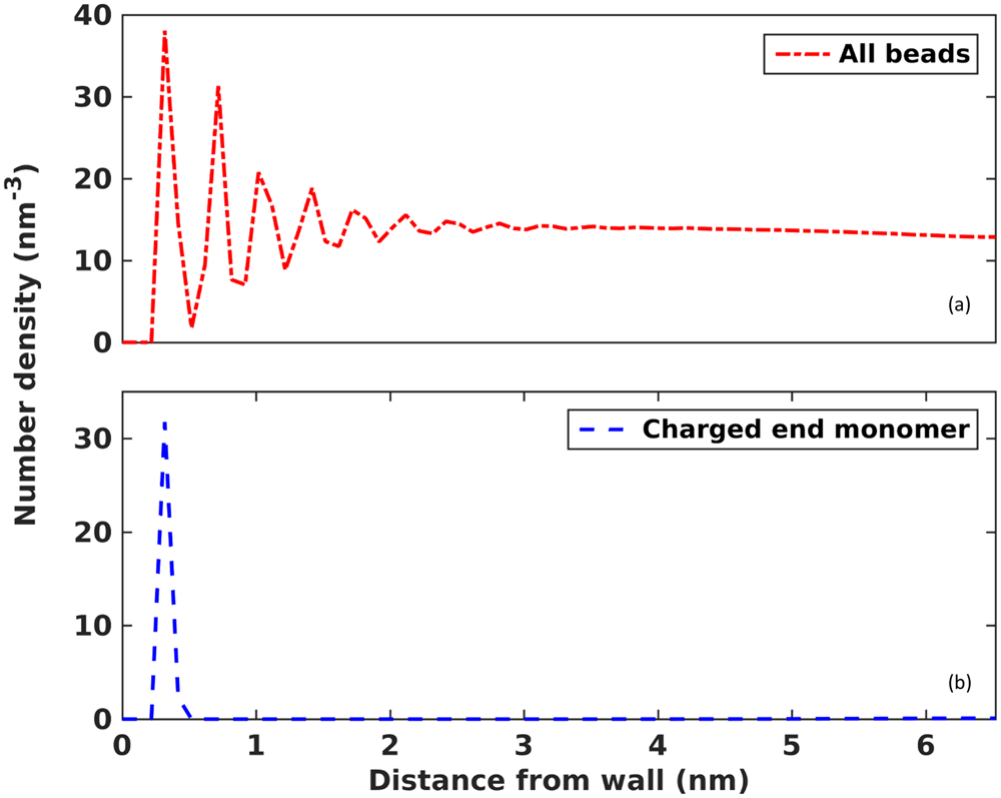
Structure of the neat canopy near the lower wall. Density profiles of all polymer beads (**a**) and charged end monomers (**b**) across the canopy.

We next examine the distribution of polymer beads, in particular the polymer’s charged end monomers, across the canopy. Figure 3a shows that the density profile of polymer beads exhibits key signatures of liquid films adsorbed on solid substrates: the bead density oscillates significantly near the wall and decays gradually as we move far away the wall. Inspection of the MD trajectories indicated that the mild interpenetration of the polymers grafted on the two walls occurs in the middle portion of the system, as anticipated based on prior simulations^12^. A key observation of the density profile shown in Fig. 3a is that, the bead density within 6 nm from the wall is slightly higher than that in bulk polymers. Such a dense packing of polymers, along with the extended-chain conformation of individual polymers revealed in Fig. 2, highlights that steep entropic cost originating from inter- and intra-polymer interactions must be overcome for the neat canopy to adopt its present structure. In our system, the only mechanism that can overcome this cost is the electrostatic attractions between the polymers’ charged end monomers and the charged wall. In prior studies of NIMs, such attractions were often conceptualized as “ionic bonds” between the polymers’ charged terminal groups and the nanoparticles’ charge sites although the long-range electrostatic interactions were computed in prior MD simulations. The interaction energy between a polymer’s charged terminal group and a charged site of a nanoparticle at contact, *E*_*ionic*_, is used to gauge the strength of the ionic bonds. With the force fields for monomers and wall atoms adopted here, the closest separation between a charged end monomer and a charged wall atom is ~0.32 nm, and thus an *E*_*ionic*_ of 13.1 *k*_B_*T* is obtained. This *E*_*ionic*_ signifies a rather modest bonding between *a single pair* of charged end monomer and charged wall atom in contact. Such an ionic “bond”, even in absence of the entropic penalty associated with inserting a polymer chain into a densely packed canopy and stretching it greatly, can be broken over a few tens of nanoseconds, thereby freeing its host polymer from the wall and allowing it to adopt random coil-like configurations. These, however, are in contrast with the canopy structure revealed in Fig. 2a and the fact that charged end monomers rarely desorb from the wall (see Section 3.2).

We suggest that the strong adsorption of the charged end monomers on the wall can be attributed to the long-range, collective electrostatic interactions between *all* charged end monomers and wall charges. This is a different perspective compared to the ionic bond concept, which emphasizes the strong electrostatic attraction between pairs of charged end monomers and wall charge site at close separation. Such a perspective is better understood by noting that the charged end monomers and the charges on wall together form an EDL, in which the long-range electrostatic interactions control the distribution of the charged end monomers (counterions). Figure 3b shows that, in the neat canopy, the charged end monomers of polymers adsorb on the wall in a single layer. The EDL formed by these monomers closely resembles the classical Helmholtz model of EDLs, in which a counterion layer condenses on the wall to screen its charge. If just 10% of these charged end monomers desorb from the wall and migrate to the canopy’s edge (say, 6 nm from the wall) to relieve their host polymers from crowding near the wall, then an electric field of ~0.6 V/nm is setup between the wall and the new position of the desorbed charged end monomers. In a neat NIM, the number of charged polymer monomers (i.e., the counterions) for each nanoparticle is equal to its net surface charge. Unless some charged monomers are shared by neighboring particles, which can occur only when the nanoparticles are small and the canopy is thin (e.g., the situation examined in ref.^12^), no extra ions are available to screen the hypothetical electric field created above. Therefore, moving each of these charged end monomers from the wall to their hypothetical positions 6 nm from the wall must overcome an electrostatic energy penalty of ~100 *k*_B_*T*. This significant electrostatic penalty thus overwhelms the entropic penalty due to polymer interactions. It explains why nearly all charged end monomers adsorb on the wall, which forces the neat canopy to adopt the structure revealed in Figs 2 and 3.

The adsorption of charged end monomers on the oppositely charged wall as a single layer rather than as a more diffusive space charge layer can also be understood using classical EDL theories. In neat canopies, the charges on the wall are screened *solely* by the charged end monomers (which serve as counterions) and there is no co-ion in the system. Such counterion-only EDLs have been studied using the classical Poisson-Boltzmann equation, and analytical solution of the ion density near walls is available. In particular, the counterion density on the surface of an isolated planar wall, *ρ*_*s*_, is given by^25^

1 $${\rho }_{s}={\sigma }_{e}^{2}/2{\epsilon }_{0}{\epsilon }_{r}{k}_{{\rm{B}}}T$$

where *σ*_*e*_ is the surface charge density on the wall and *ε*_0_ is the vacuum permittivity. In presence of additional charged walls, *ρ*_*s*_ near individual walls increases. Using parameters relevant to our system (*σ*_*e*_ = −3.5 *e*/nm^2^, *ε*_*r*_ = 10, *T* = 400 K), a *ρ*_*s*_ of 322 nm^−3^ is obtained. Such a *ρ*_*s*_ exceeds the close packing density of the charged end monomers, and is caused by the fact that the finite ion size is not taken into account in the Poisson-Boltzmann equation. Nevertheless, it indicates that there is a strong tendency for all charged end monomers to adsorb on the wall. To see this more quantitatively, let’s assume that 90% of the surface charge of the wall is neutralized by end monomers contact-adsorbed on the wall, and consider how the rest of the charged end monomers distribute in the EDL. Here the wall’s effective surface charge density is reduced to −0.35 *e*/nm^2^, and Eq. 1 predicts a density of *ρ*_*s*_ = 3.2 nm^−3^ for the charged end monomers on the wall. If we assume that these monomers occupy a layer thickness of 0.1 nm, the surface density of these monomers is 0.32 *e*/nm^2^, which is about the same as the effective surface charge density of the wall. Therefore, these monomers will be localized within a few angstroms from the wall.

A key premise of the above discussions leading to the idea that charged end monomers form a compact, rather than diffusive, layer near wall, is that co-ions are absent from the system. If co-ions exist in the system, then the thickness of an EDL is characterized by the Debye length *λ*_*D*_ = (*ε*_0_*ε*_*r*_*k*_B_*T*/2*e*^2^*ρ*_∞_)^1/2^ in 1:1 dilute electrolytes, where *ρ*_∞_ is the counter/co-ion density in the bulk solution, i.e., outside of the EDL (in electrolytes such as RTILs, *λ*_*D*_ is not a good indicator of the EDL thickness, and theories^26,27^ that can take into account the ion-ion correlations are needed). As ion contamination is difficult to completely remove in NIM, co-ions likely exist in NIMs and affect the EDL thickness (or more specifically, how the charged end monomers distribute near the wall). It is safe to expect that the charged monomers of some polymers will be “exchanged” off the charged walls by the added counterions and thus their host polymers are no longer “grafted” on the walls. Nevertheless, a quantitative picture is not yet available and some questions remain open. For example, is amount of polymers desorbed from the wall equal to the number of added counterions? How do the added counterions and co-ions distribute in the canopy? What conformation do the desorbed polymers adopt? To answer these questions, we next study canopies doped with electrolyte ions.

#### Doped canopies

We again first focus on the conformation of individual polymers. When pairs of Na^+^ and Cl^−^ ions are introduced into the canopy, the counterions (Na^+^ ions) adsorb on the wall. They adsorb on the wall more easily and strongly than the charged end monomers of the polymers because, unlike the charged end monomers, their adsorption does not incur entropic penalty associated with the stretching of polymer chains. Since these Na^+^ ions screen part of the wall charges, they displace some of the charged end monomers originally adsorbed on the wall, causing their host polymers to become “free” polymers (see Fig. 2b). Trying to avoid the entropic penalty due to polymer crowding near the wall, these “free” polymers migrate away from the wall and adopt conformation close to those of bulk polymers. For example, as shown in Table 2 and Fig. 2c, their mean *R*_g_ and *L*_ee_ as well as the distribution of their *L*_ee_ are all nearly indistinguishable from those of bulk polymers.

In the doped canopy, the charged end monomers of many polymers are still associated with the charged wall (though more dynamic than that in the neat canopy, see Section 3.2), thus attaching them to the wall. On average, ~90 charged end monomers are associated with each wall, which gives an effective “grafting” density of *σ* ≈ 1.41 nm^−2^. The much lower effective grafting density compared to that in neat canopy means that the crowding of polymers near the wall is greatly relieved in the doped canopy. Figure 2b suggests that, while these wall-associated polymers are still stretched, the stretching is no longer significant. In fact, their *R*_g_ and *L*_ee_ are only ~10% and 20% larger than those of bulk polymers, respectively (see Table 2); the peak position of the distribution of their *L*_ee_ is only shifted by 1 nm from that in bulk polymers (see Fig. 2c). Using the *σ* and *R*_g_, obtained above, the reduced “grafting” density is found to be ${\rm{\Sigma }}=\sigma \pi {R}_{g}^{2}\approx 5.45$. Because this Σ is larger than 5, the typical cutoff for wall-grafted polymers to be considered as polymer brushes^28^, the wall-associated polymers may be considered as polymer brushes. However, we emphasize that these wall-associated polymers are not statically grafted to the wall as in conventional polymer brushes, and they can explore a rather large configurational space. For example, Fig. 2d shows that the distribution of their *θ*_ee-w_ is not narrowly focused near 0° as in the neat canopy and a weak peak even appears at *θ*_ee-w_ ≈ 90°. The latter indicates that both the charged and neutral end monomers of a polymer can adsorb on the wall simultaneously, which rarely occurs in the neat canopy.

**Figure 4:**
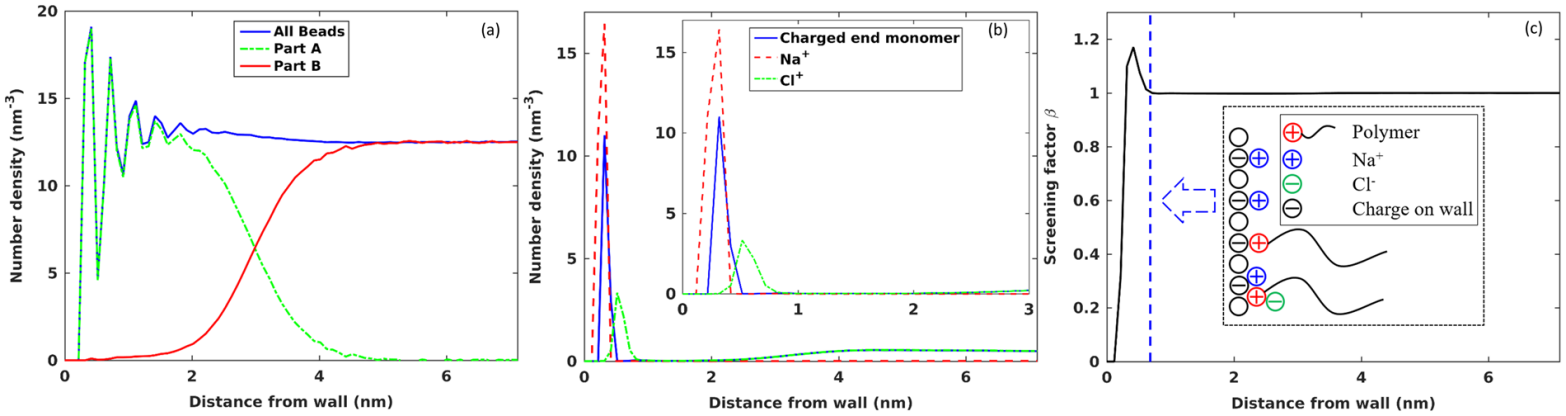
Structure of the doped canopy in direction normal to the solid wall. (**a**) Density profile of the polymer beads near the charged solid wall and its contribution by polymers whose charged end monomer resides in different regions (Part A: *z* = 0–0.6 nm; Part B: *z* > 0.6 nm). (**b**) Density profiles of the charged end monomers, Na^+^, and Cl^−^ ions. (**c**) Variation of the screening factor *β* near the charged solid wall. Inset shows the arrangement of polymer and electrolyte ions near the wall.

Overall, doping a canopy with electrolyte ion pairs causes some polymers originally “grafted” to the wall by electrostatic interactions to detach and become “free” polymers. This greatly relieves the crowding of polymers near the wall, and allows both the free and grafted polymers in the canopy to adopt more random coil-like conformations, which should change the average structure of the canopy. To assess such change, we next compute the polymer density profile across the doped canopy. Figure 4a shows that the polymer bead density in doped canopy is lower than that in neat canopy. Meanwhile, the distance between the two walls increases. Since the space between these walls is shared by the two canopies near them, we define the canopy thickness as half of the equilibrium separation between the top and bottom walls. The thickness of the neat and doped canopies is found to be 6.47 ± 0.01 nm and 7.10 ± 0.01 nm, respectively. Hence, doping leads to a dilation of the canopy by ~10%.

The above discussion shows that the response of a neat canopy to doping by Na^+^/Cl^−^ ion pairs depends strongly on the adsorption (desorption) of Na^+^ ions (charged end monomers) from the wall, or more generally the change of the EDL structure in the canopy. To quantitatively assess the change of the EDL structure caused by electrolyte ion doping and to gain more insight into the structure of doped canopies, we examine the EDL structure in the doped canopy.

Figure 4b shows the density profiles of the Na^+^ ion, Cl^−^ ion, and the charged end monomer across the canopy. The Na^+^ ions form a single layer near the wall. Some charged end monomers, having a size larger than the Na^+^ ions, adsorb on the wall to form another distinct layer. These counterion layers are followed by a co-ion (Cl^−^ ion) layer. The arrangement of these ion layers are shown schematically in the inset of Fig. 4c. To explore how these ion layers screen the charge on the wall, we compute the charge screening factor *β* as a function of distance from the wall^29^

2 $$\beta (z)=-1/{\sigma }_{e}\,{\int }_{0}^{z}{\rho }_{e}(s)\,ds$$

where *ρ*_*e*_(*s*) is space charge density due to ions and charged end monomers at a distance *s* from the wall, and *σ*_*e*_ is the surface charge density on the wall (−3.5 *e*/nm^2^). At positions with *β* > 1.0, the surface charge on the wall is over-screened. Figure 4c shows that overscreening occurs in the region 0.3 nm < *z* < 0.75 nm, and disappears at *z* > 0.75 nm. Examining *ρ*_*e*_ and its components due to various species shows that the adsorption of Na^+^ ions on the wall screens ~75% of its charge. However, these Na^+^ ions displaces 59.8% (rather than 75%) of the charged end monomers *originally* adsorbed on the wall, i.e., Na^+^ ions do not simply execute an ion exchange with the charged end monomers adsorbed on the wall. Consequently, more counterions adsorb on the wall than that is needed to fully screen its charge, and overscreening occurs. Such an overscreening brings Cl^−^ ions toward the wall (see the Cl^−^ peak at *z* ≈ 0.52 nm) to neutralize the excessive charges due to Na^+^ ions and charged end monomers adsorbed on the wall. Hence overscreening disappears at *z* = 0.75 nm. Overscreening originates from the strong ion-ion correlations in electrolytes^27^ and is a generic feature of EDLs in RTILs^27,29,30^. NIMs can be viewed as unique member of RTILs^1^, with the nanoparticles, charged polymers, and the introduced ion pairs as ions. As such, overscreening is rather expected. In molecular RTILs, overscreening affects the capacitance-voltage curve of the EDLs in them^27^. Here, by reducing the electrolyte doping-induced desorption of charged end monomers, overscreening enhances polymer crowding in the interfacial region.

At *z* > 0.75 nm, the wall charge is fully screened. For EDLs in conventional electrolytes, the densities of counterions and co-ions are equal *and* uniform beyond this position. Here, the densities of the charged end monomers (counterions) and Cl^−^ ions (co-ions) are indeed close to each other at *z* > 0.75 nm. However, their distributions are highly non-uniform: they are depleted in the regions centering *z* ~ 1.5 nm and accumulate preferentially in the regions *z* > 4 nm. Such non-uniform distribution is mainly caused by the entropic interactions between polymers. For example, the region centering at *z* ~ 1.5 nm is populated by polymers whose charged end monomers adsorb on the wall (see Fig. 4a, in which the density of the beads of these polymers are shown as green dashed line). Inserting a charged end monomer (and hence its host polymer) here incurs a large entropic penalty. Hence few charged end monomer appears here. Since local electro-neutrality is maintained at *z* > 0.75 nm, Cl^−^ ions are also depleted here.

#### Discussion

The canopy structure revealed above is obtained from a system that best resembles polymeric canopies in NIMs with large nanoparticles, moderately long polymer chains, and moderate to high surface densities on the particle surface. The canopy structure revealed here agrees broadly with the onion-like model derived from prior experimental data. In particular, the observation that, in doped canopies, there can be two populations of polymers, some anchored to the nanoparticle’s surface/corona and the rest interacting weakly with nanoparticles, is captured in our simulations. In addition, the fact that doping using electrolyte ions increases the population of polymers interacting weakly with nanoparticles is also reproduced. Our simulations further suggest that these polymers form more than one layers near the charged solid surface and there is notable energy barrier (as implied by the valleys between the density peaks of the charged end monomers in Fig. 4b). Therefore, the structure of polymeric canopy truly resembles that of an onion in that there are many layers of polymers in the canopy. The energy barrier for polymers in different layers should affect the exchange of the polymers between these layers and thus can impact the transport properties (e.g., electrical conductivity) of NIMs.

Some difference between the simulation results and experimental observations does exist. In neat canopies, the existence of polymers that are not “grafted” to charged walls cannot be established with certainty in our simulations. This is in contrast to the suggestion by prior NMR measurements that some polymers in NIMs are not “grafted” to nanoparticles even in absence of intentionally added electrolyte ions^3^. The difference is likely caused by two reasons. First, the molecular model adopted here may have underestimated the strength of the entropic penalty of stretching the polymers. For example, since polymers are modeled as flexible chains, our model may underestimate the energy penalty of stretching a non-flexible polymer chain. Second, the nominally neat canopies probed experimentally may contain electrolyte ion pairs. NIMs are often prepared by concentrating dilute solutions, and thus impurities presented in nominally pure solutions are extremely difficult to remove. Indeed, some studies showed that even fully neutralized NIMs contain ~0.1Na^+^ ions per charged site on the nanoparticle’s surface^2,3,16^.

### Canopy dynamics

#### Polymer exchange dynamics

**Figure 5:**
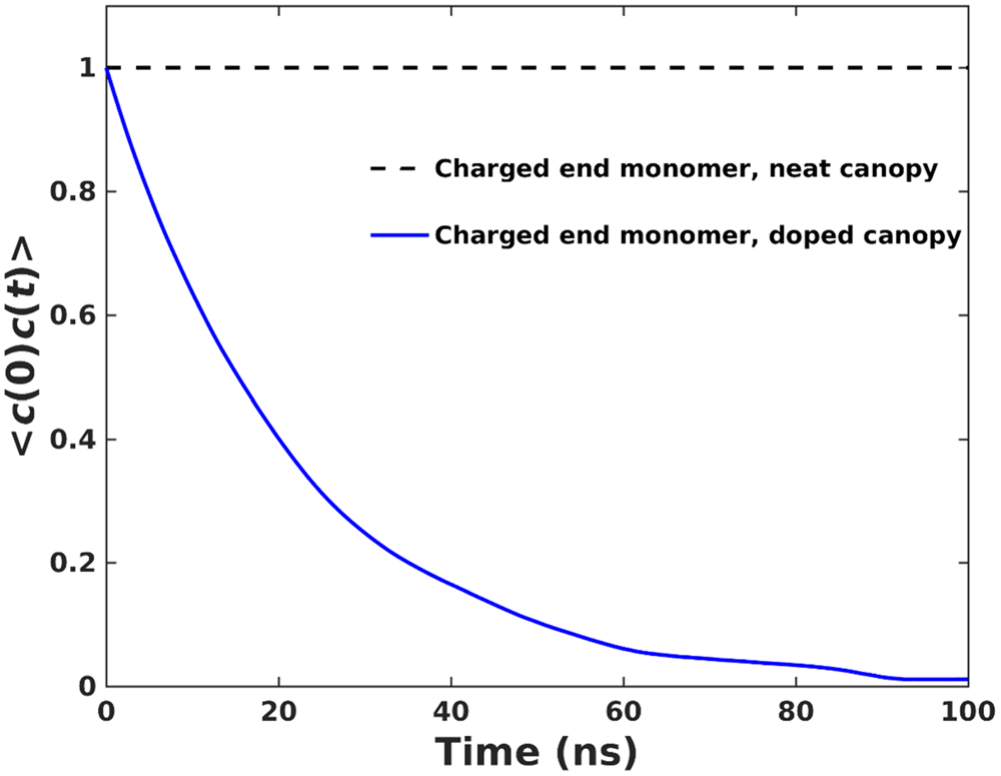
Time correlation function for the adsorption of a charged end monomer on the solid wall. A charged end monomer is considered to be associated with the wall if it is within 0.6 nm from the wall (i.e., in the first density peak of the charged end monomer in Fig. 4b).

**Figure 6:**
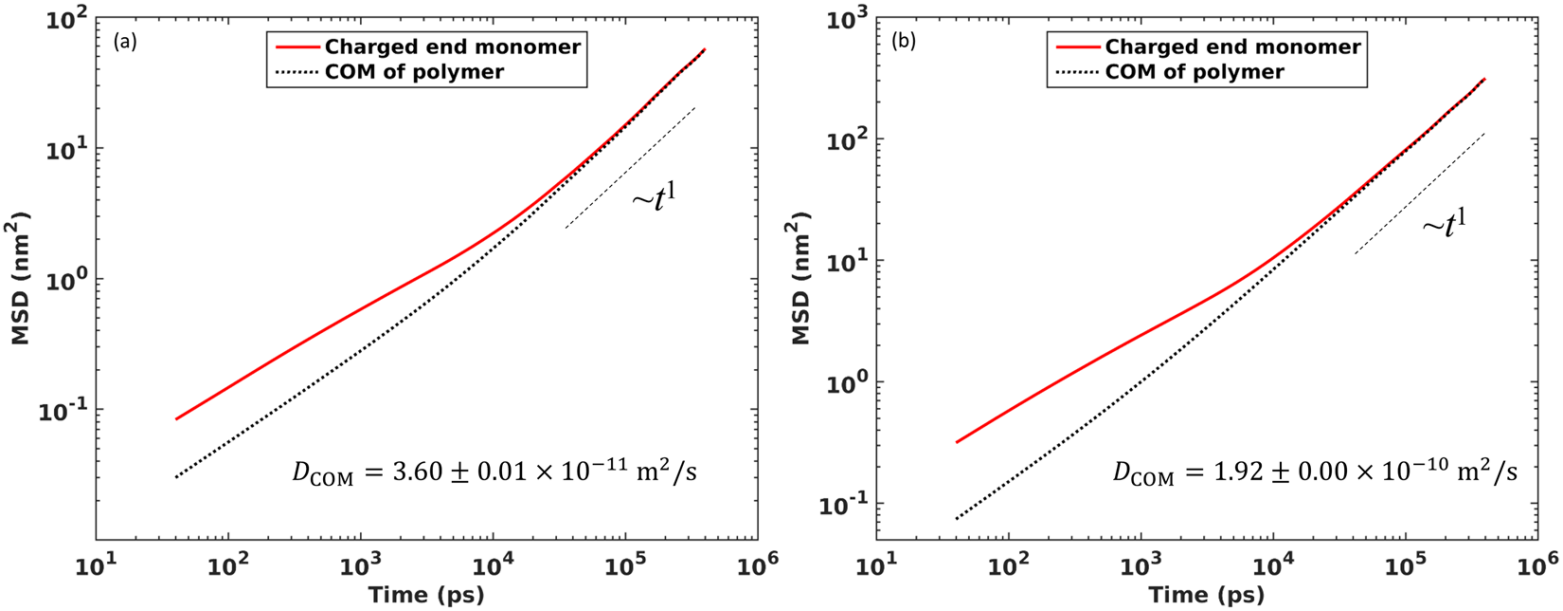
Diffusion of polymers in neat and doped canopies. The lateral mean-square-displacement (MSD) of the charged end monomer and center-of-mass (COM) of polymers in (**a**) the neat canopy and (**b**) the doped canopy.

To assess how fast a polymer grafted electrostatically to the charged walls desorb and exchange with other “free” polymers (if exist) in the canopy, we compute the time correlation function for the association of a charged end monomer with the charged wall

3 $$AC{F}_{c}(t)=\langle c(0)c(t)\rangle $$

where *c*(*t*) is defined as 1.0 if a charged end monomer is within 0.6 nm from the wall (see Fig. 4b), and 〈…〉 denotes ensemble average. Figure 5 shows that, in neat canopies, the adsorbed charged end monomers do not desorb over the sub-microsecond time scale probed in our simulations. This is consistent with the very high free energy penalty for a counterion (charged end monomer here) to move away from the wall in absence of co-ions. This result is different from that obtained from simulation of NIMs with particle diameter of 1 nm in ref.^12^, where it was found that a polymer can hop from one nanoparticle to another nanoparticle over hundreds of nanoseconds. This difference is in fact anticipated because the hopping of polymer from one particles to another in the prior study was enabled by the bridging of two charged nanoparticles by the charged monomers in the polymers. Because such bridging does not occur in our systems, once a polymer is “grafted” to one of the charged walls, it rarely leaves that wall. Interestingly, we find that the charged monomer attached to one the charged walls can diffuse from one charged site on the wall to other sites (this is also evident from the modest lateral diffusion coefficient of the attached polymers on the wall, see Fig. 6). This is consistent with the finding from previous simulations that the charged monomer of a polymer grafted dynamically to a nanoparticle can move between different charged sites of the same nanoparticle^12^.

Figure 6 shows that, in doped canopies, desorption of the charged end monomers from wall occurs at a time scale of one hundred nanoseconds. This is consistent with the experimental observation that, in NIMs, the exchange between the free and bound polymers in canopies is greatly accelerated by doping the NIMs using electrolyte ions^3^.

#### Single polymer dynamics

We examine the diffusion of polymers in directions parallel to the wall by computing their mean-square displacement (MSD) in the *xy*-plane. Figure 6a shows that, in neat canopies, despite that the charged end monomers of each polymer is adsorbed strongly on the wall and do not desorb over sub-microsecond time scale, these monomers exhibit modest in-plane mobility. Such a phenomenon can be attributed to the hoping of the charged end monomers between the charged sites on the same solid surface, which has also been reported in a prior MD study of NIMs consisting of 1 nm-radius nanoparticles and charged polymers^12^. Figure 6a shows that, at short time scale (<20 ns), the MSD of the center-of-mass (COM) of polymers is distinctly smaller than that of the charged end monomers. This is expected. At short time, the movement of the charged end monomers is hindered mostly by electrostatic interactions with charged wall atoms in contact with them. The movement of a polymer’s COM, however, is the net result of the movement of all its beads, and is thus additionally hindered by other factors such as entanglement of polymer chains. At short time, the MSDs of both the charged end monomer and the COM exhibits sub-diffusive behavior as can be expected for diffusion in a crowded environment. The sub-diffusive movement of the charged end monomer persists to a longer time than the COM, likely because the movement of these monomers is strongly arrested by the attractions by the charged wall atoms. From the MSD shown in Fig. 6a, the average lateral diffusion coefficient of polymers’ COM is determined to be 3.60 ± 0.01 × 10^−11^ m^2^/s, which is about an order of magnitude slower than that of the bulk polymers (*D*_*bulk*_ = 3.16 × 10^−10^ m^2^/s).

Figure 6b shows the MSDs of the charged end monomers and the COM of the polymers in doped canopies. Over the same time period, these MSDs are much larger than their counterparts in the neat canopies. This is caused by two reasons. First, there are many “free” polymers in the doped canopies and these polymers exist in an environment rather similar to that in bulk polymers, which allows fast diffusion. Second, there is relatively fast exchange between the “free” polymers and the polymers “grafted” to the charged wall. Using the MSD shown in Fig. 6b, the average lateral diffusion coefficient of polymers’ COM is determined to be 1.92 ± 0.00 × 10^−12^ m^2^/s, which is moderately smaller than bulk polymers but ~5 times faster than the polymers in neat canopies. This result is in good agreement with the observation that doping canopies using electrolyte ions greatly accelerates the diffusion of the polymers in them^3^.

## Conclusions

In summary, we study the polymeric canopies in model NIMs with moderate polymer chain length, relatively high surface charge density, and in the limit of large nanoparticle diameter. In neat canopies, charged end monomers form a Helmholtz layer near the charged surface, thereby “grafting” practically all polymers to the wall. These polymers are highly stretched. While hardly desorbing from the wall, they maintain modest lateral mobility. Doping the canopy with electrolyte ions causes some of the polymers to migrate away from the wall and become “free” polymers. The remaining “grafted” polymers are no longer highly stretched and the “free” polymers adopt configuration similar to bulk polymers. Therefore, the canopy is dilated. The “free” polymers move in an environment similar to that in bulk polymers. This, along with the rather rapid exchange between the “grafted” and “free” polymers, means that polymer dynamics in the canopy is greatly accelerated by electrolyte doping.

The canopy structure and dynamics revealed in our simulations are consistent with those inferred from prior experimental studies. However, some subtle features not yet widely recognized are also noted. For example, while the desorption of the charged end monomers from the wall is driven by the adsorption of electrolyte counterions introduced into the canopy, the number of the desorbed monomers is less than that of adsorbed electrolyte counterions due to the overscreening phenomenon.

The structure and dynamics of polymeric canopies as well as their response to electrolyte doping are controlled primarily by the interplay of electrostatic and entropic effects. Adjacent to the charged wall, electrostatic effects dominate over the entropic effects, and polymers are “grafted” to the wall despite significant entropic penalty associated with polymer crowding. Beyond this interfacial layer, entropic effects play a more important role, which leads to the formation of multiple polymer layers. While the importance of electrostatic interactions is long recognized, until now attention has been focused on interactions between *individual* charged monomers and charged wall atoms. However, we show that the long-range, *collective* electrostatic interactions are important in deciding the canopy structure when the wall’s surface charge density is high. In this regard, it is instructive to consider the canopy as a special electrical double layer screening the charges on the wall. While entropic effects due to polymer crowding undoubtedly give new characteristics to these double layers compared to those in RTILs, some key features of double layers in RTILs such as overscreening are preserved. Such a perspective of the canopies in NIMs complements the “ionic bond” concept adopted in prior models for NIMs, and may help develop improved theories for NIMs.

## Electronic supplementary material

Supplementary information
