# Supplementary material for: Structure and Dynamics of Polymeric Canopies in Nanoscale Ionic Materials: An Electrical Double Layer Perspective
Source: Sci Rep. 2018 Mar 26;8:5191. doi: 10.1038/s41598-018-23493-1 (PMC5979949; doi:10.1038/s41598-018-23493-1)
Supplement: Supplementary file 1 — Supplementary information [file 41598_2018_23493_MOESM1_ESM.pdf]

Supplementary Information

Structure and Dynamics of Polymeric Canopies in Nanoscale Ionic Materials: An Electrical Double Layer Perspective

Zhou Yu,[1] Fengchang Yang,[1] Sheng Dai,[2] and Rui Qiao[1,*]

[1] *Department of Mechanical Engineering, Virginia Tech, Blacksburg, Virginia 24061*

[2] *Oak Ridge National Laboratory, Bethel Valley Road, Oak Ridge, Tennessee 37831*

1. Force field parameters for polymers, ions and wall atoms

All atoms are modeled as charged/neutral Lennard-Jones (LJ) particles. The potential ϕ_{ij} for the non-bonded interaction between a pair of atoms *i* and *j* is given by

$$\phi_{ij}(r) = 4\epsilon_{lj,i-j}\left[\left(\frac{\sigma_{lj,i-j}}{r}\right)^{12} - \left(\frac{\sigma_{lj,i-j}}{r}\right)^{6}\right] + \frac{1}{4\pi\epsilon_r\epsilon_0 r}$$

where r is the separation between the two atoms. $\epsilon_{lj,i-j}$ and $\sigma_{lj,i-j}$ are the LJ parameters for the atom pair. ϵ_0 is the vacuum permittivity and ϵ_r is the dielectric constant (taken as 10 here). Table S1 lists the charge and the LJ parameters for each atom type. The LJ parameters for atom pair (*i*, *j*) are obtained from those of the atom pairs (*i*,*i*) and (*j*,*j*) using the Lorentz-Berthelot combination rule.

Table S1. Lennard-Jones parameters and atom charge for atoms in the MD system

	$\boldsymbol{\sigma_{lj,i-i}}$ (Å)	$\boldsymbol{\epsilon_{lj,i-i}}$ (kJ/mol)	charge (e)
**wall atom**	3.386	2.4470	0 or -1
**polymer bead**	4.000	3.1320	0 or +1
**Na+**	2.876	0.5216	+1
**Cl^-**	3.785	0.5216	-1

* Corresponding author. Email: ruiqiao@vt.edu.

2. The evolution of polymer chain's conformation in neat canopy system

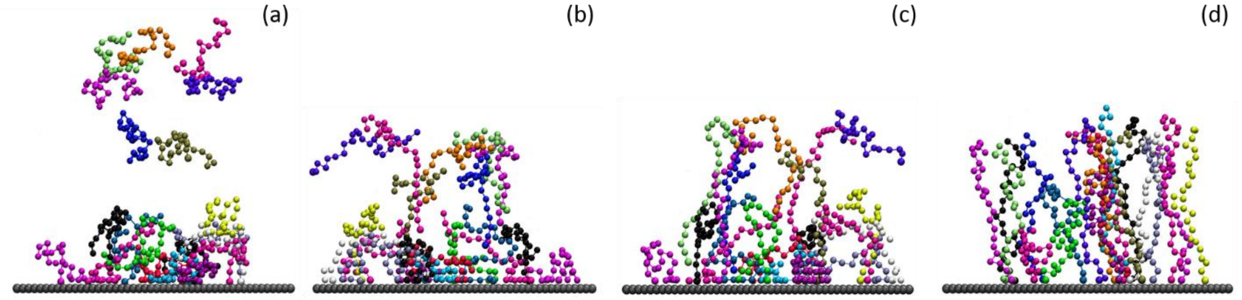

Figure S1. *Evolution of polymer chains' conformation in the neat canopy system during the equilibration process*. Initially, polymers with random, coil-like conformation are packed between the two charged walls. The snapshots of 20 representative polymers near the lower wall are shown at time 0 ps, 400 ps, 800 ps, and 10 ns in panels (a), (b), (c), and (d), respectively.
